# Bridging the gap between CVD and COVID-19: the oxidized LDL hypothesis

**DOI:** 10.3389/fmed.2025.1588062

**Published:** 2025-08-06

**Authors:** Jalil Daher

**Affiliations:** Department of Biology, Faculty of Arts and Sciences, University of Balamand, El-Koura, Lebanon

**Keywords:** COVID-19, endothelial dysfunction, atherosclerosis, cardiovascular disease, dyslipidemia, HDL, LDL, oxidized LDL

## Abstract

Severe acute respiratory syndrome coronavirus 2 (SARS-CoV-2) is an enveloped positive sense RNA virus and the causative agent of COVID-19. The viral envelope includes the spike (S) glycoprotein which mediates the entry of the virus to the host cell. The S protein comprises the receptor binding domain (RBD) that is responsible for binding to the angiotensin-converting enzyme 2 (ACE 2) receptor on the surface of target cells. ACE 2 is highly expressed on the endothelium lining blood vessels which may explain the cardiovascular symptoms of COVID-19 patients. Emerging evidence suggests that COVID-19 and cardiovascular disease (CVD) may share important mechanisms that regulate their pathogenesis and that endothelial dysfunction (ED), which has been already shown to be tightly linked to CVD, plays an instrumental role in the pathogenesis of COVID-19 by mainly affecting the hyperinflammatory and coaguloatory states that are seen during disease progression. Meanwhile, there is also increasing evidence suggesting that COVID-19-linked ED is due to a dysregulation in lipid metabolism pathways. Of note, it has been reported that low high density lipoprotein (HDL) levels correlate with the severity of COVID-19 through a potential impairment in the antioxidant capacity of HDL which may lead to lipid oxidation and the generation of oxidized low density lipoprotein (LDL). Interestingly, we have previously shown that myeloperoxidase oxidized LDL (Mox-LDL) possesses an anti-fibrinolytic activity in endothelial cells; this may negatively affect the course of COVID-19 by increasing the chance of complications such as disseminated intravascular coagulation events and ischemic strokes. In this article, I hypothesize that targeting inflammatory dyslipidemia could be highly beneficial in treating COVID-19 patients and improving their clinical outcome.

## Introduction

Severe acute respiratory syndrome coronavirus 2 (SARS-CoV-2) is a member of the Coronaviridae and Coronavirinae family and subfamily of viruses, respectively. It is an enveloped positive sense RNA virus and the causative agent of COVID-19 (1). The viral envelope contains multiple components that include the membrane (M) and envelope (E) proteins as well as the spike (S) glycoprotein which is instrumental during the first steps of infection when the virus binds to its target receptor to gain entry to the host cell (2). The S protein, more specifically its S1 subunit that includes the receptor binding domain (RBD), is responsible for binding to the angiotensin-converting enzyme 2 (ACE 2) receptor in order to promote the cell entry of the virus (3). Of note, the virus binding target ACE 2 is a crucial player in the renin-angiotensin-aldosterone system (RAAS), where it is responsible for the conversion of Angiotensin II (AngII) into Ang 1–7, which leads to a decrease in blood pressure and inflammation (4). On the other hand, the S protein can activate immune cells such as macrophages by binding to the membrane toll-like receptors TLR2 and TLR4 leading to the release of potent pro-inflammatory mediators such as IL-6 and TNF-α, contributing to the cytokine storm that is seen in severely ill patients with COVID-19 (5). The distribution of the ACE 2 receptor in different tissues may elucidate the variability in COVID-19 patients' symptoms. For instance, the replication and release of the virus into the lung tissue can lead to multiple symptoms that include headache, fever, respiratory complications and myalgia (6). In the intestines and kidneys, the presence of the ACE 2 receptor can lead to gastrointestinal and kidney complications after infection with SARS-CoV-2. Notably, ACE 2 is highly expressed in endothelial cells that line blood vessels which may explain the cardiovascular symptoms and complications that are reported in COVID-19 patients (7). In fact, postmortem examination of the heart tissue of patients who died of COVID-19 has shown severe endotheliitis and myocardial infarction. Much remains unsolved especially that it is becoming clearer nowadays that cardiovascular disease (CVD) and COVID-19 share many important pathological mechanisms that regulate their onset and progression (3, 8). The questions remain whether virus-driven inflammation and endothelial dysfunction (ED) leading to coagulopathy may contribute to a higher risk of ischemic infarcts in COVID-19 patients and whether CVD risk factors, mainly dyslipidemia, may have analogous reciprocal implications during the course of infection? These questions will require further research to better clarify the hypotheses behind them. This article will focus on the hypothesis that describes the potential involvement of dyslipidemia and oxidized LDL in the dysfunctional endothelial state that is seen during SARS-CoV-2 infection as well as in the symptoms of COVID-19.

## COVID-19 pathogenesis

COVID-19 has been associated with many complications that include severe vascular endotheliitis, coagulopathy, systemic inflammation, and “cytokine storm” (9). It has been shown that most COVID-19 non-survivors exhibit nonspecific infection markers that are linked to cardiac injury as well as blood coagulation abnormalities (10). Meanwhile, emerging evidence suggests that endotheliitis plays an instrumental role in the pathogenesis of COVID-19 since it leads to ED that is followed by the hyperinflammatory and deregulated hemostasis phenomena that are seen during disease progression. On the other hand, it has been already shown that ED is tightly linked to CVD which is considered as a principal comorbid factor that affect the survival of patients with COVID-19 (11). Interestingly, it has been reported that COVID-19 strongly correlates with ischemic strokes in patients where the virus triggers a cascade of thrombotic events after entering to the bloodstream during the course of infection (12, 13). The attachment of the virus to the ACE 2 receptor on the surface of endothelial cells and its internalization leads to ACE 2 depletion which result in Angiotensin II (AngII) accumulation, NADPH activation and the increase in reactive oxygen (ROS) generation, oxidative stress and overall ED (11, 14). The latter plays a detrimental role during SARS-CoV-2 infection by fueling inflammation and blood clotting, thus, multiplying the risk of disseminated and excessive intravascular coagulation which lead to an increase in the rate of fatalities and a poor prognosis in COVID-19 patients (11, 15). Meanwhile, ED is also responsible for the amplification of the expression of major pro-inflammatory cytokines such as TNF-α, IL-1β and IL-6 that further enhance blood clotting through the downregulation of anticoagulant and pro-fibrinolytic factors such as thrombomodulin and the upregulation of anti-fibrinolytic factors that are responsible for the reduction in fibrinolysis and the initiation of blood clotting pathways (16–18). Lastly, there is increasing evidence suggesting that ED, which is accompanied by drastic changes and alterations in inflammatory responses and hemostatic mechanisms in COVID-19 patients, is also due to a dysregulation in lipid metabolism pathways (3).

## Dyslipidemia and COVID-19

When managing CVD, the measurement of plasma levels of lipids and lipoproteins is very essential and critical in assessing cardiovascular risk (Table 1). For instance, a low level of high-density lipoprotein (HDL) cholesterol is used as a biomarker and an important predictor for increased risk of myocardial infarction and CVD complications (19, 20). HDL is conventionally known to promote reverse cholesterol transport from the peripheral tissue to the liver (3). Besides its latter well-known function, HDL is also involved in various mechanisms that are tightly linked to the modulation of immune responses during infection. For instance, HDL can bind to pathogen associated lipoteichoic acids and lipopolysaccharides dampening the immune response. In addition to their immunomodulatory activities, HDL particles were reported to possess antithrombotic and antioxidant effects (21). Interestingly, it has been lately suggested that there exists a relationship between low levels of HDL cholesterol and the risk of an infectious hospitalization (22). More specifically and in the context of COVID-19, it was recently reported that low levels of HDL cholesterol correlate with the severity of the disease although the mechanism behind it is not yet clearly defined (23). It has been speculated that the inflammatory burden that is seen during SARS-CoV-2 infection affects the composition of the apolipoprotein fraction of HDL. For instance, inflammation may lead to drastic changes in the expression of apoliprotein genes in the liver and may also promote the interaction between pro-inflammatory proteins such as serum amyloid protein A and HDL particles causing the displacement of ApoA-1, altering HDL function and further intensifying the inflammatory response (24). Meanwhile, oxidative stress is highly involved in modifying HDL and increasing its dysfunction by inactivating its antioxidant enzyme paraoxonase 1 (PON1). Subsequently, impaired HDL antioxidant capacity may result in a vicious cycle of lipid oxidation which could generate oxidized low density lipoprotein (LDL) and aggravate inflammation and tissue damage (25). Also, remarkably and in the context of COVID-19, it was recently shown that the long-term disease symptoms in women are associated with lower cholesterol levels regardless of age or patient history (26). On a similar note, studies have intriguingly shown a decline in LDL cholesterol levels during SARS-CoV-2 infections. Despite the absence of any evidence linking this decrease to COVID-19 outcomes, possible reasons for those low LDL levels might be related to inflammation that can induce liver injury, interfering with LDL synthesis, and, at the same time, increase its modification and its uptake my macrophages which confirms the present hypothesis (27, 28). Notably, besides the reduction in cholesterol levels, recent clinical studies have shown an increased oxidative stress that is linked to lipid peroxidation in patients with COVID-19 (29). Interestingly, it has been demonstrated that SARS-CoV-2 infection is associated with an increased oxidation of LDL particles which can have deleterious effects on disease progression (30). Meanwhile, it has been also reported that the modification of LDL particles in COVID-19 patients induces phenotypic changes in LDL that correlate with some adverse and poor clinical outcomes (31). Moreover, it has been shown that oxidized LDL and arterial stiffness are strongly linked to the post-acute complications of COVID-19 (32). On this particular note, it has been reported that circadian rhythm-associated pathways that are tightly linked to oxidative stress and CVD regulate post-acute sequelae of COVID-19 (33).

**Table 1:** Major human plasma apolipoproteins and their functions.

**HDL**	**LDL**	**Oxidized LDL**
– Reverse cholesterol transport of excess cholesterol from the cells to the liver. – Immunomodulatory activity during infections. – Anti-inflammatory action. – Anti-thrombotic effects. – Antioxidant capacity.	– Delivery of cholesterol from the liver to cells.	– Activation of endothelial dysfunction. – Pro-atherogenic capacity. – Pro-inflammatory effects. – Pro-thrombotic action. – Anti-fibrinolytic activity. – Pro-oxidant.

HDL, high-density lipoprotein; LDL, low-density lipoprotein.

In addition to that, it has been shown that patients with severe COVID-19 exhibit higher levels of oxidized LDL that correlates with disease severity (34). Given the available clinical data, it seems evident that oxidized LDL plays an essential role in COVID-19; accordingly, it has been demonstrated that, along with its major scavenger receptor LOX-1, this modified form of LDL may represent a reliable marker in the diagnosis of COVID-19 (35).

## The modification of LDL

LDL is considered as the major vehicle responsible for the transport of cholesterol in the circulation. Inflammation and oxidative stress can modify LDL particles into their pro-athrogenic form that play a crucial role in the initiation and progression of atherosclerosis, the culprit behind CVD (36). Atherosclerosis is a chronic inflammatory condition that involves vascular endothelial cells as well as various immune cells, namely macrophages and their derived foam cells, which are considered as the hallmark of the disease. In atherosclerosis, the vascular endothelium becomes inflamed and dysfunctional due to the effect of multiple insults including oxidized LDL that accumulate in the subendothelial space of the intima prompting the mechanism of arterial remodeling and the thickening of the arterial wall (37). During this process, macrophages are responsible for the internalization of oxidized LDL particles culminating in the formation of the so-called “foam cells” which contribute to the development of atherosclerotic lesions (36). Among the various factors that are linked to atherosclerosis, the oxidation of LDL remains the most significant risk factor because of the versatile role that it may play during the evolution of the disease. Particularly, LDL that is modified by myeloperoxidase has been proposed as the most patho-physiologically relevant form of oxidized LDL that reflects what happens *in vivo* during the process of atherogenesis (38). Remarkably, it has been shown that the level of oxidized phospholipids is significantly increased in the lungs of patients during viral infections (39). Meanwhile, oxidized lipids such as oxidized LDL are recognized by scavenger receptors that are expressed on many types of cells including macrophages, endothelial cells and smooth muscle cells; consequently, the latter types of cells could be involved in maintaining the inflammatory responses and pathways that are detected in different pathological states including atherosclerosis. Of note, the lectin-like oxidized LDL receptor (LOX-1) is considered as a major type of scavenger receptors and its role in ED and atherosclerosis is very well documented. LOX-1 binds to oxidized LDL leading to the induction of pro-inflammatory and pro-oxidant pathways contributing to atherosclerotic plaque development (40, 41). Interestingly, it has been reported that LOX-1 may be activated during SARS-CoV-2 infection and may play an instrumental role in the pathogenesis of COVID-19. It has been recently reported that LOX-1 may be implicated in acute lung injury and Kawasaki-like multisystem inflammation seen during COVID-19 progression (42, 43).

## Mox-LDL pathways in ED: implications for COVID-19

We have previously shown that myeloperoxidase oxidized LDL (Mox-LDL) is associated with ED where it is responsible for sustaining inflammatory pathways that enhance atherosclerosis. We have studied the effect of Mox-LDL on various endothelial cell functions and have reported that this oxidized form of LDL has the ability to interfere with wound healing and angiogenesis *in vitro*. In this particular context, we have shown that Mox-LDL may increase dysfunction at this level by activating signaling pathways that involve microRNA-22 and heme oxygenase-1 (44). In addition, we have investigated the effect of Mox-LDL on pro-atherogenic mechanisms in macrophages where we have demonstrated its significant role in switching macrophages into a pro-inflammatory state and increasing lipid accumulation in them leading to foam cell formation (45, 46). Similarly, we have reported that Mox-LDL enhances inflammation in endothelial cells through the activation of NF-κB and increase in IL-8 secretion; the latter is considered as an instrumental factor during the evolution of the atherosclerotic plaque. Interestingly, we were able to link Mox-LDL's pro-inflammatory phenotype to LOX-1 activation (47). Meanwhile, we have also shown that Mox-LDL may possess an anti-fibrinolytic activity in endothelial cells which may have many implications in the context of atherosclerosis and COVID-19. In atherosclerosis, a decrease in fibrinolysis may play a negative role during the evolution of the disease by enhancing fibrin deposition on the endothelial lining of blood vessels leading to ED and inflammation and culminating in an increase in the permeability of the endothelium to lipid infiltration enhancing the process of atherogenesis. This will also increase blood clotting and the chance of myocardial infarction and stroke (48, 49). In this particular context, we have shown that Mox-LDL regulates the process of fibrinolysis in endothelial cells through LOX-1 signaling and the modulation of neuroserpin expression. The latter is a member of the serine proteinase inhibitor superfamily, with similar activities to plasminogen activator inhibitor-1 (PAI-1), having a major function in inhibiting tissue plasminogen activator (tPA) in the brain. During the process of fibrinolysis, tPA is secreted by endothelial cells where it is responsible for the conversion of plasminogen into plasmin and the reduction in blood clotting. Of note, tPA has been reported to be very effective in the treatment of patients with acute thrombotic complications (50–52). As already mentioned, there is growing amount of data for COVID-19 being a disorder of blood clotting where the viral infection is tightly linked to complications that include disseminated intravascular coagulation events and ischemic strokes (11–13). It is speculated that the downregulation of endothelial ACE 2 that is induced by SARS-CoV-2 infection may result in an increase in oxidative stress and the transformation of β2-glycoprotein 1 into its oxidized form that can no longer inhibit the procoagulatory capacity of the von Willebrand factor (vWF) produced by the dysfunctional endothelium (53, 54). The latter factor is also amplified by the action of a plethora of pro-inflammatory mediators, such as IL-6, IL-1β and TNF-α which make part of the “cytokine storm” that is seen during complicated infections and that is associated with a poor prognosis and increased fatalities in patients with COVID-19 (11). Notably, those pro-inflammatory cytokines may play an additional important role in the upregulation of anti-fibrinolytic factors, namely PAI-1, decreasing tPA activity and altering blood hemostasis which culminate in an increased risk of thrombosis in affected patients. Overall, I hypothesize that Mox-LDL may play an instrumental role in this particular context by similarly affecting tPA levels in patients with COVID-19 which contributes to higher thrombotic incidences and fatalities.

## Conclusion

Overall, no drug has proven to be completely efficient for treating the complications of COVID-19. Multiple monoclonal antibody therapies that were aimed at suppressing the “cytokine storm” by individually blocking some major pro-inflammatory cytokines such as IL-1 or IL-6 were thoroughly investigated with limited success (3). Other therapeutic agents that has been recently assessed as potential treatment strategies for COVID-19 due to their possible effects in mitigating inflammatory reactions are still preliminary and require further testing in relevant clinical trials. For instance, initial *in vitro* and pilot randomized, double-blind, placebo-controlled studies have shown that Diacerein could have some beneficial effects in terms of COVID-19 therapy due to its role in decreasing viral replication by downregulating the activation of the inflammasome (55). Similarly, additional preclinical data involving the ACE inhibitor Lisinopril has proven to be inconsistent demonstrating contrasting effects in drug-treated mice that have shown higher viral loads which was accompanied by a heightened anti-inflammatory state (56).

**Figure 1:**
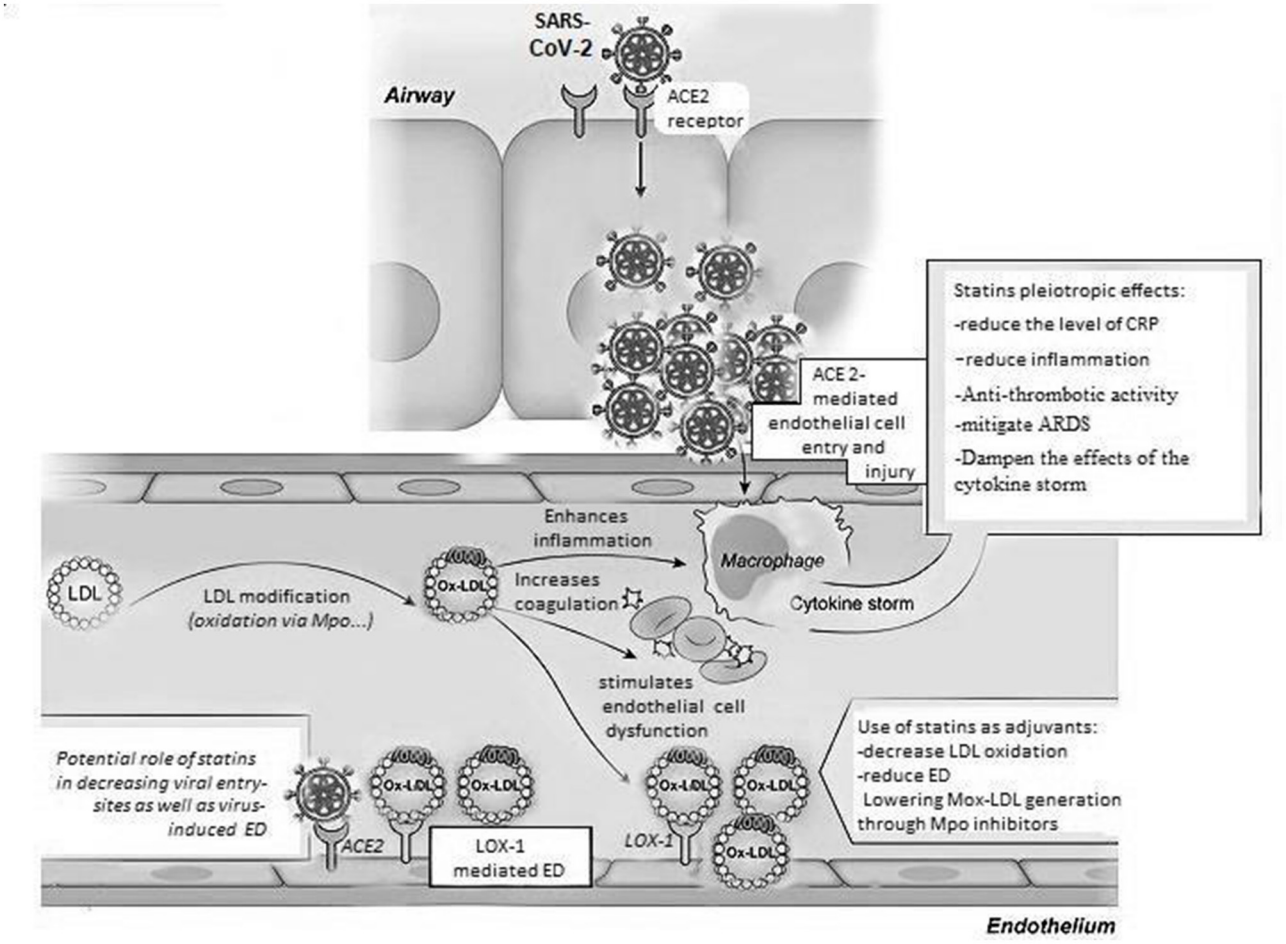
Oxidized LDL pathways in COVID-19. Multiple pathways, including modification by the Myeloperoxidase enzyme, can lead to oxidized LDL generation. The latter form of LDL induces various mechanisms that may affect the progression of COVID-19; those mechanisms include the induction of pro-inflammatory pathways inside of macrophages and endothelial cells enhancing the cytokine storm and endothelial dysfunction that are driven by the SARS-CoV-2 virus. More importantly, this oxidized version of LDL could be possibly involved in increasing coagulation contributing to excessive blood clotting which leads to a significant rise in the rate of fatalities and a poor prognosis in COVID-19 patients. Lipid-lowering therapies including statins used for the treatment of vascular events in CVD could be potentially useful as adjuvants in the management of COVID-19 because of their pleiotropic effects that involve a decrease in LDL oxidation and LOX-1 scavenger receptor-mediated ED, as well as systemic inflammation and thrombosis, mitigating ARDS and the effects of the cytokine storm seen during the late complications of COVID-19. ACE 2, angiotensin converting enzyme 2; LDL, low density lipoprotein; Ox-LDL, oxidized LDL; Mox-LDL, myeloperoxidase oxidized LDL; ED, endothelial dysfunction; LOX-1, lectin-like oxidized LDL receptor; CRP, C reactive protein; ARDS, acute respiratory distress syndrome; SARS-CoV-2, severe acute respiratory syndrome corona virus 2.

In this article, we hypothesize that targeting inflammatory dyslipidemia, including LDL level and quality, could be highly beneficial in treating COVID-19 patients and improving their clinical outcome. Essentially, the “cytokine storm” that underlies COVID-19 leads to low HDL cholesterol levels as well as an increase in LDL oxidation. These lipid irregularities could be treated by pharmacological agents that decrease the level of LDL in patients and limit its oxidation. On the other hand, these pharmacological interventions should be tailored to increase HDL plasma level as well as its APOA-1 content. Lipid-lowering therapies including statins are considered as the most common agents that are used for the treatment of both primary and secondary vascular events (57). Apart from their primary mechanism of action involving the inhibition of 3-hydroxy-3-methylglutaryl-CoA reductase (HMGCR) as well as the increase in HDL and cholesterol uptake by cells, statins are believed to possess additional pleiotropic effects, including antithrombotic and anti-inflammatory activities, just to name a few (58). Accordingly, statins have been examined as promising adjuvants in the treatment of viral infections (59). Indeed, the use of statins in COVID-19 is a subject that is worth investigating due their impending benefits in this particular context. Of note, statins have been shown to reduce the level of CRP and the generation of oxidized LDL (28, 60, 61). They have been also reported to reduce inflammation in the endothelium by alleviating ED. In addition, lowering LDL results in the reduction of membrane bound cholesterol and the sites of viral entry into target cells (61). Also, it has been recently reported that statins may play an important role in mitigating the effects of acute respiratory distress syndrome that is seen during the late complications of COVID-19; in fact, it has been shown that statin administration to patients with COVID-19 during hospitalization results in a significant reduction in mortality rates (62–64). Meanwhile, there exist a debate regarding which statin could be used as an additional therapeutic agent in service of COVID-19 treatment. On this account, some types of statins are being reconsidered nowadays to target oxidized LDL-driven inflammatory pathways; for example, Simvastatin has been rediscovered lately to play a role in regulating oxidized LDL-induced pro-inflammatory mechanisms in macrophages. In addition, several drugs, including the antimicrobial and antioxidant drug benzoic acid hydrazide, were identified to reversibly inhibit MPO activity which could hold a great promise for future therapeutic interventions aimed at lowering LDL oxidation (50). Meanwhile, the therapeutic benefits of human cathelicidin antimicrobial peptide LL-37 is a subject warranting careful investigation; it has been documented that LL-37 is highly involved in the regulation of neutrophils extranuclear traps (NETs) dynamics and combatting SARS-CoV-2 infection. Of a particular relevance to the present topic, one mechanism could implicate the clearance of MPO that is abundant within NETs, potentially altering LDL oxidation and ameliorating COVID-19 symptoms (65). Also, other HDL-raising drugs that could be considered for the treatment of COVID-19 include CETP-inhibitors, fibrates and small molecules that increase APOA-1 synthesis (66). By ultimately inhibiting the coagulation cascade, the latter interventions could be highly beneficial in treating patients for chronic atherogenic responses pertaining to CVD while counteracting the negative impact of the short term complications that are seen during COVID-19 progression (Figure 1). Any drug used as an adjunctive therapy in the treatment of COVID-19 will need, however, careful testing and close monitoring for possible drug interactions or adverse reactions for establishing its safety and efficacy. Ultimately, the necessity for testing of lipid-lowering drugs and carefully assessing the cardiovascular history in patients with COVID-19, including dyslipidemia and LDL oxidation, by prioritizing clinical approaches that measure Mox-LDL levels in patients, is a subject that requires further examination and scrutiny. Accordingly, additional clinical data as well as specifically designed studies are needed in order to confirm the various speculations and hypotheses that revolve around the subject. Thus, it is urgently required nowadays that as much research data as possible could be collected for the development of more appropriate guidelines for the proper diagnosis and treatment of COVID-19 in patients with cardiovascular history, enabling the scientific community to retrieve more impactful information regarding the interplay between COVID-19 and the cardiovascular system.

## Data Availability

The original contributions presented in the study are included in the article/supplementary material, further inquiries can be directed to the corresponding author.
